# ChromoMapperWeb: evaluate genome alignments and track assembly steps within an interactive graphic environment

**DOI:** 10.1093/nar/gkag506

**Published:** 2026-05-21

**Authors:** Elvira Toscano, Elena Cimmino, Angelo Boccia, Leandra Sepe, Giovanni Paolella

**Affiliations:** CEINGE—Biotecnologie Avanzate “Franco Salvatore”, Via Gaetano Salvatore, 486, Napoli 80145, Italy; Dipartimento di Medicina Molecolare e Biotecnologie Mediche, Università degli Studi di Napoli Federico II, Via Sergio Pansini, 5, Napoli 80131, Italy; CEINGE—Biotecnologie Avanzate “Franco Salvatore”, Via Gaetano Salvatore, 486, Napoli 80145, Italy; Dipartimento di Medicina Molecolare e Biotecnologie Mediche, Università degli Studi di Napoli Federico II, Via Sergio Pansini, 5, Napoli 80131, Italy; CEINGE—Biotecnologie Avanzate “Franco Salvatore”, Via Gaetano Salvatore, 486, Napoli 80145, Italy; Dipartimento di Medicina Molecolare e Biotecnologie Mediche, Università degli Studi di Napoli Federico II, Via Sergio Pansini, 5, Napoli 80131, Italy; CEINGE—Biotecnologie Avanzate “Franco Salvatore”, Via Gaetano Salvatore, 486, Napoli 80145, Italy; Dipartimento di Medicina Molecolare e Biotecnologie Mediche, Università degli Studi di Napoli Federico II, Via Sergio Pansini, 5, Napoli 80131, Italy

## Abstract

Rapid quality assessment and multiple assembly comparison are essential steps while assembling new genomes or re-sequencing known ones. Many available tools used for assembly evaluation produce global metrics, representing assembly quality or overall features, most of them working as command line tools that typically act on large data files and produce long detailed result files, where it is not always easy to identify regions of similarity or difference among different chromosome assemblies. ChromoMapperWeb is a new web tool that takes as input nucmer or QUAST output files, quickly identifies similarities and differences between the compared assemblies, and displays them using both table visualizations and pre-arranged or custom graphics. Graphical displays are interactive and allow progressive zoom levels which, in a few steps, move from full genome to very enlarged views, where even small alignment blocks are easily identified. The program, freely accessible through the web server https://chromomapperweb.ceinge.unina.it/, provides an easy-to-use graphical interface, used for experiment planning and interactive evaluation of the results, which include tables and graphical representations of whole genomes, chromosomes, or single blocks.

## Graphical Abstract

**Graphical Abstract:**
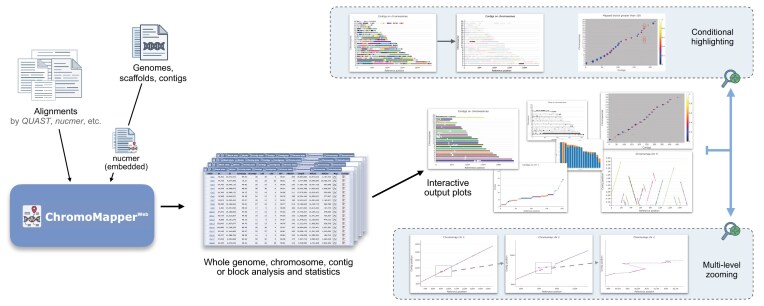

## Introduction

Quality assessment and assembly comparison are essential steps while sequencing new genomes or evaluating the structure and organization of a previously assembled genome. Over the years, many assembly-evaluation tools following different approaches have been developed and made available to the scientific community. Some methods are reference-free and map reads onto the final assembly to check consistency and detect errors or estimate the completeness of an assembly by looking for known features, such as conserved genes [1–7] or repeated genomic regions [8]. Reference-based tools, on the other hand, compare one or more assemblies with a reference genome: many of them depend on one of the available genome sequence aligners, such as nucmer, included in MUMmer system [9, 10], or minimap2 [11]. Aligners typically produce raw files organized as lists of alignment blocks, subsequently evaluated to provide synthetic parameters; these result files contain much information but it is not easy to quickly identify major regions of similarity or main differences between the compared assemblies. The MUMmer package has for a long time provided such information and is also used as a base by other tools to further process its output. More recently, QUAST, originally designed for bacterial and small eukaryotic assemblies and later upgraded to support large genomic sequences [12, 13], became a popular tool, often used to evaluate a large range of metrics. It supports comparison against a user-provided reference genome and is very effective at finding small as well as large similarities between compared assemblies. Some of this information is made available in graphic format taking advantage of Icarus [14], a browser integrated within QUAST to complement the output with aligned block visualization. QUAST has recently been made available within a web server, WebQUAST, to allow remote execution and result browsing through a graphical interface [15].

Overall, these tools are very good at rapidly distinguishing low-quality assemblies from good ones, but intermediate assemblies are often less easy to evaluate. Differences between similar genomes are sometimes easier to spot with the help of visualization tools [16, 17]; examples are MGView, a simple early one, originally designed to support gap closure of microbial genomes [18] or Synteny Portal [19], which can visualize synteny blocks by using prebuilt alignments in the UCSC Genome Browser database [20]. More recently, support for synteny tracks and structural variations among genomes has been also included in full featured genome browsers, like JBrowse2 [21], or comparative genomic viewers, like CGV [22], although, this last is currently limited to display pre-built whole-genome alignments provided by NCBI rather than newly assembled genomes. A recent command line tool, ChromoMapper, directly uses nucmer or QUAST output files to quickly identify and display similarities and differences between assemblies [23]. It supports the comparison of assemblies produced by short- or long-read sequencing [23, 24] and can reach a good level of detail.

Here, we present a new web tool, ChromoMapperWeb, which improves on assembly visualization and interpretability of alignment results. It works by importing genome alignment files produced by QUAST or nucmer and helps navigate through alignment blocks, quickly identifying and displaying alignment regions at genome, chromosome, contig, or block scale. The program takes a spreadsheet-like approach and displays alignment data both in tables containing synthetic or detailed reports and in zoomable graphic maps. ChromoMapperWeb shares data analysis code with the command line tool ChromoMapper [23], but largely improves on it by providing an easy-to-use graphical interface for experiment planning and interactive result selection and evaluation, and adds the option to upload assembled genome sequences and align them by using nucmer. The program is made available at https://chromomapperweb.ceinge.unina.it/; it is free and open to all users and there is no login requirement.

## Materials and methods

### Overview of ChromoMapperWeb workflow

ChromoMapperWeb uses the mapping of a genome assembly onto a reference one to evaluate similarities and differences between compared genomes or assemblies and to display them in both table and graphic representations. Most code relies on PHP programming language (version 8.2). It uses an object-oriented approach and takes advantage of library functions and interface objects previously developed in the laboratory [23, 25, 26] for basic data management, table building, and web page output.

The alignment blocks produced during the alignment phase are post-processed to relate reference and assembly sequences at different levels: contigs, scaffolds, or fully assembled chromosomes. An overview of the whole process is presented in Supplementary Fig. S1; the main aspects regarding alignment production and import of the results are described in detail in the next paragraphs, while further details are included in Supplementary Text S1.

### Alignment of genome assemblies

ChromoMapperWeb uses as input genome alignments provided in different formats. Result folder produced by running QUAST on assembled sequences may be uploaded either as a zip archive containing the whole folder or as single selected files. A .tsv file is used to optionally provide names and lengths of the reference chromosomes. Nucmer from the MUMmer package may also be used to produce alignment files that are directly read; results from other tools may need to be modified before import. Result files may also be imported by sharing them through a local web server and providing a URL link to them.

ChromoMapperWeb can also calculate simple genome alignments by running nucmer on assemblies provided by the users. Alignments involving small genomes, composed of one or few chromosomes, are run immediately, while larger tasks including mammalian genomes are scheduled according to available resources and become analysable when ready. The program supports alignments in three different modes: ‘same genome’, used for alternative assemblies of the same sequences or to evaluate the results of the different steps of the assembly procedure; ‘same species’, used for genome re-sequencing of new individual genomes; and ‘close species’, for example, for different species genome comparison. These three modes essentially differ for three alignment parameters: minimum single match length (-l), maximum gap between two adjacent matches in a cluster (-g), and distance an alignment extension will attempt to extend poor scoring regions before giving up (-b), respectively, set to 100, 90, and 500 for same genome, 60, 90, and 1500 for same species, and 40, 120, and 1200 for close species. The raw output file (delta file) produced by nucmer is then processed and converted into a .tsv file by two additional tools of the same package, delta-filter and show-coords, which filter out alignment blocks shorter than 500 bases (-l 500), or with identity (-i) <95% (same genome, same species) or 92% (close species).

### Management of nucmer alignment tasks

Alignments involving small genomes, composed of one or few chromosomes, are usually run immediately, while larger tasks including mammalian genomes are scheduled according to available resources and become analysable when ready. The execution of nucmer-based alignment tasks within the web server is managed through an asynchronous queue system described in detail in Supplementary Text S1.

### Import of alignment results

The import phase produces a data structure (the ‘chromoRunner’ object), which is used to store the information provided by the user during the experiment setup, organized into alignment run data, such as date and run name, number of alignment experiments, and used mapping tool. For each alignment experiment, a ‘chromoExp’ object is built to store experiment-specific data, such as alignment blocks, name of reference genome and mapped assembly, assembly features, and reference genome chromosome data. These data are provided by the user or, whenever possible, extracted from the alignment result files. During the import phase, alignment blocks are taken from the provided .tsv file, re-formatted taking into account differences between QUAST and nucmer formats, and stored in the chromoExp object. Reference genome features, such as chromosome number and lengths, are obtained from the uploaded .tsv file, if this is available. Alternatively, the import module produces an estimate of the length of the reference assembly sequences from the leftmost block start and rightmost block end positions present in the alignment files, sorts them by descending size, and assumes that sequences corresponding to chromosomes are those >50% of the previous one.

If a QUAST result folder is used as source, alignment experiments are automatically read from the files in the ‘contigs_reports’ folder (the user can always modify them later) and reference genome chromosomes, if not provided as .tsv file, are guessed using the lengths obtained from the genome_info.txt in the ‘genome_stats’ folder. Similarly, assembly features, including total length, number of contigs, and L90, L75, L50, N90, N75, and N50 values, are also automatically extracted from the report.tsv file.

### Web pages and graph production

**Figure 1.**
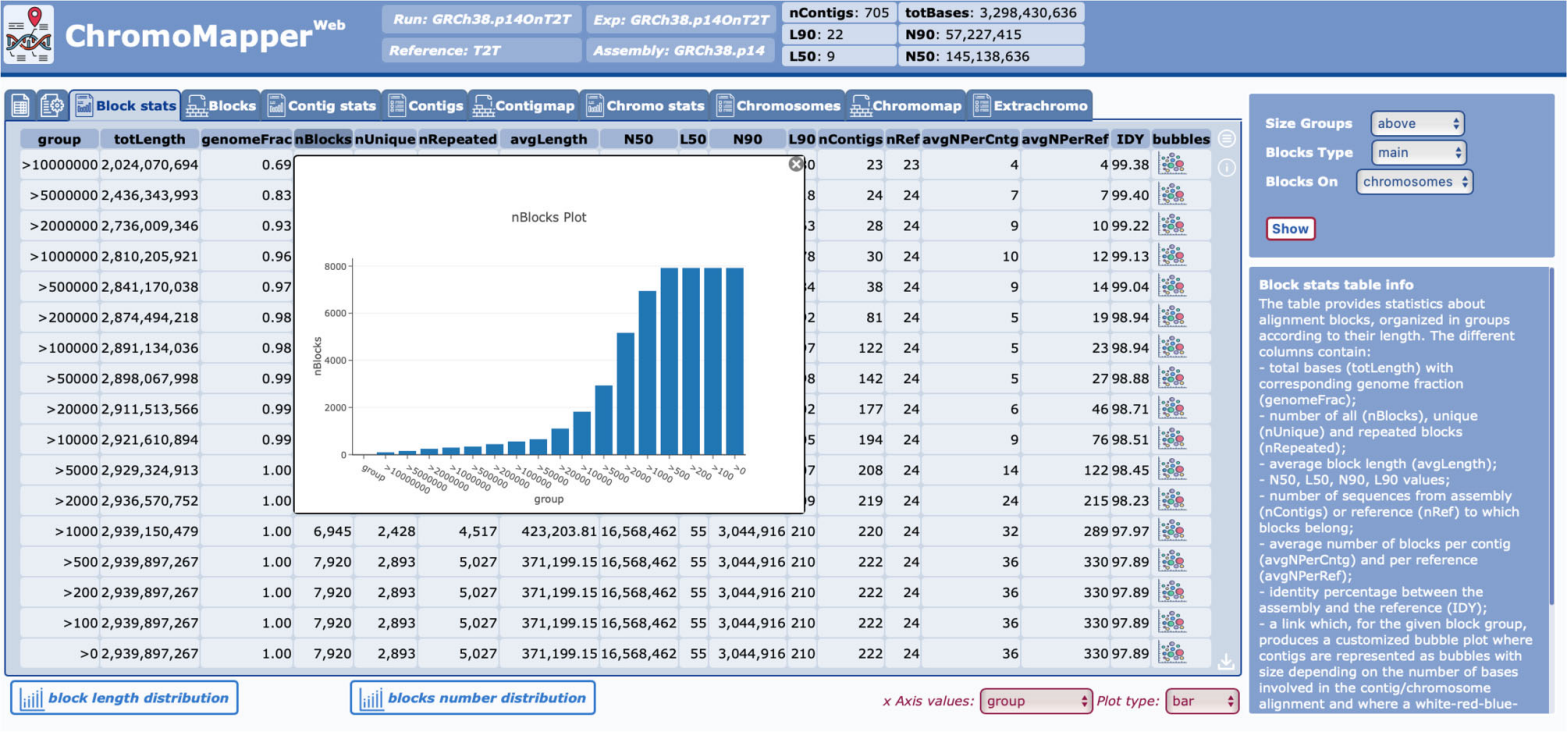
Web page containing alignment statistics in table format. The results are normally provided as spreadsheet like tables, organized under different tabs. Links give access to graphics. The graph over the table is a custom plot, produced on the fly by plotting values in column ‘nBlocks’ against column ‘group’.

ChromoMapperWeb uses a web-based interface to allow users to submit data, set up experiments, and visualize results in an organized way. The home page gives access to forms where users can upload input datasets, define alignment, and process settings. The web server employs a label-based mechanism that, without requiring user registration, enables the logical aggregation of related analyses by associating experiments with a user-defined tag. The results section uses multiple, dynamically created interactive tables, organized in a spreadsheet-like structure, where users can browse data and build plots on the fly, selecting any column as the *x*- or *y*-axis (Fig. 1). Tables can be interactively re-calculated, by using the form to change parameters.

ChromoMapperWeb produces a variety of plot types with interactive features, such as zooming to study the results at various levels of detail. Plots take advantage of the plotly library and its ability to use JavaScript to produce dynamic plots in the browser window and to remain fast even in the presence of large amount of data [23, 26, 27]. All interactive actions are executed on the client side to allow for fast response, even when visualization becomes more complex. As most plots use colour, to make data easier to read from users with impaired colour vision, colour palettes are provided, specific for the main types of colour-vision deficiency, in addition to a grey-scale palette. An advanced interactive system has been setup to make it easier to highlight data points sharing the same feature. In bubble plots, for example, hovering over a bubble highlights, with red circles, bubbles from the same contig that maps on different chromosomes. Similarly, in contigs on chromosomes plot, clicking on a block can highlight those from the same contig, through coordinated opacity changes and synchronized hover effects.

### Source of genome assemblies and production of QUAST alignments

External genome alignments were obtained by QUAST [12, 13], version 5.0.2, by defining the parameters ${--\!}$*eukaryote* and ${--\!}$*large* to indicate that the genome is eukaryotic, not circular and >100 Mb, in order to have an effect on speed and accuracy by the imposition of ${--\!}$*min-contig 3000*, ${--\!}$*min-alignment 500*, ${--\!}$*extensive-mis-size 7000* and allowing to identify misassemblies caused by transposable elements and excluding them from the number of misassemblies.

For *Staphylococcus aureus*, assemblies produced by ABySS [28], ALLPATHS-LG [29], Bambus2 [30], SGA [31], SOAPdenovo [32], and Velvet [33] were taken from Salzberg *et al*. [34]. All the other sequences were taken from NCBI website: GCF_000013425.1, i.e. reference genome sequence for *S. aureus*; GCA_011064465.1 and GCA_011064465.2, i.e. 1.7 and 2.2 versions for the individual human genome [35]; GCA_040939325.1, GCA_040939355.1, GCA_021951015.1, GCA_021950905.1, GCA_050656345.1, and GCA_050656315.1, i.e. SNU1272, HG002-LCL, and RPE-1 cell line phased genome assemblies [36–38]; GCF_009914755.1, i.e. the human telomere-to-telomere (T2T) assembly [39, 40]; GCF_028858775.2, GCF_037993035.2, and GCF_006542625.1, i.e. assemblies for *Pan troglodytes, Macaca fascicularis*, and *Nomascus leucogenys* genomes, respectively [41, 42]. The HG002-LCL name is used to indicate an early passage immortalized lymphoblastoid cell line (LCL) derived from B lymphocytes from one human sample (HG002) [37].

The synteny plots, between human chromosome 1 and macaque and gibbon chromosomes, were produced by using the pairwise genome alignment tool available on the Ensembl website (https://www.ensembl.org/).

## Results

### Comparing alternative assemblies of the same read set generated by different tools

**Figure 2.**
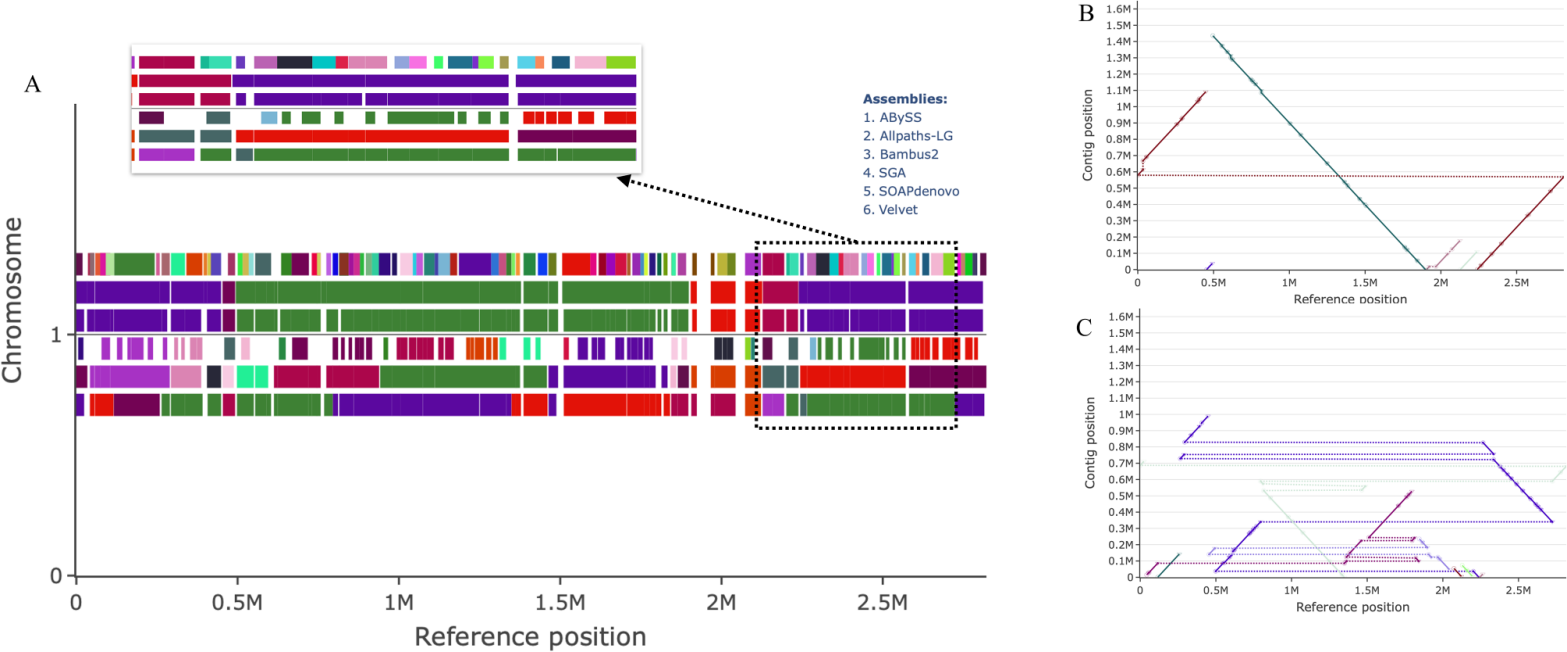
Alignment of assemblies produced by different tools from a set of *S. aureus* reads. Assemblies produced by ABySS, ALLPATHS-LG, Bambus2, SGA, SOAPdenovo, and Velvet aligned with *S. aureus* genome sequence (GCF_000013425.1) used as reference. (**A**) Multitrack genome plot, where alignment blocks are reported as coloured rectangles according to the contig they belong to and located at the position on which they map on the reference chromosome (Mb); the arrow indicates a zoom on the region 2.1–2.7 Mb. (**B** and **C**) Assemblies by ALLPATHS-LG and Velvet, aligned with the reference sequence, are reported as dotplot-like graphs, where blocks are represented by segments placed at their reference/contig position (Mb) and tagged with start (circles) and stop (triangles) symbols. Dotted lines are used to connect non-contiguous blocks on the reference chromosome.

Comparative evaluation of assemblies produced starting from a given read set is often a desirable option when choosing between alternative assembly procedures or when comparing the performance of different tools. Figure 2A shows such an example where six assemblies of *S. aureus* genome, produced by different tools starting from a common read set [34], are displayed side by side and aligned with a reference sequence, the fully assembled genome obtained from NCBI website (GCF_000013425.1). The first assembly, by ABySS [28], is clearly the most fragmented one, while the second, by ALLPATHS-LG [29], is the most continuous and very similar to the third, Bambus2 [30], even though the latter presents a higher number of blocks. The assembly by SGA [31] consists of only 21 contigs but is the most patchy with many uncovered areas, while last two assemblies, by SOAPdenovo [32] and Velvet [33], show intermediate fragmentation levels. The two identical gaps around position 2 Mb, present in all the alignments, most likely correspond to real differences between reference and sequenced genomes, while other differences in the 3′ end (enlarged) are more likely due to the assemblers as they tend to be different from each other.

The different qualities of the second and the sixth alignments, both including long contigs, are clearly visible in Fig. 2B and C, where the alignments are reported in detail as a dot-like representation. ALLPATHS-LG (Fig. 2B) is composed of only five contigs, two of them (1.1 and 1.4 Mb) long enough to cover over 70% of the reference genome. The two halves of the red contig are only apparently represented as separate blocks, mapping at the beginning and end of the reference genome; in fact, they are connected through the ends, due to the circular nature of the *S. aureus* genome. In contrast, the assembly by Velvet (Fig. 2C) consists of eight long contigs, but presents many sequence relocations, possibly pointing to a higher error level during the assembly steps. The same plots are reported for all assemblies in Supplementary Fig. S2, together with a second graph that provides a contig-by-contig representation of alignment blocks, plotted as rectangles at the position on which they map on the reference chromosome. Taken together, the two plots allow a quick assessment of the quality of the assembled chromosome.

### Evaluating chromosome continuity while sequencing an individual genome

Two different versions, 1.7 and 2.2 (GCA_011064465.1 and GCA_011064465.2 from NCBI website), of a human individual genome [35] were aligned to the current human T2T assembly [39, 40] to test the ability of ChromoMapperWeb in comparing different assembly levels of a given individual genome.

**Figure 3.**
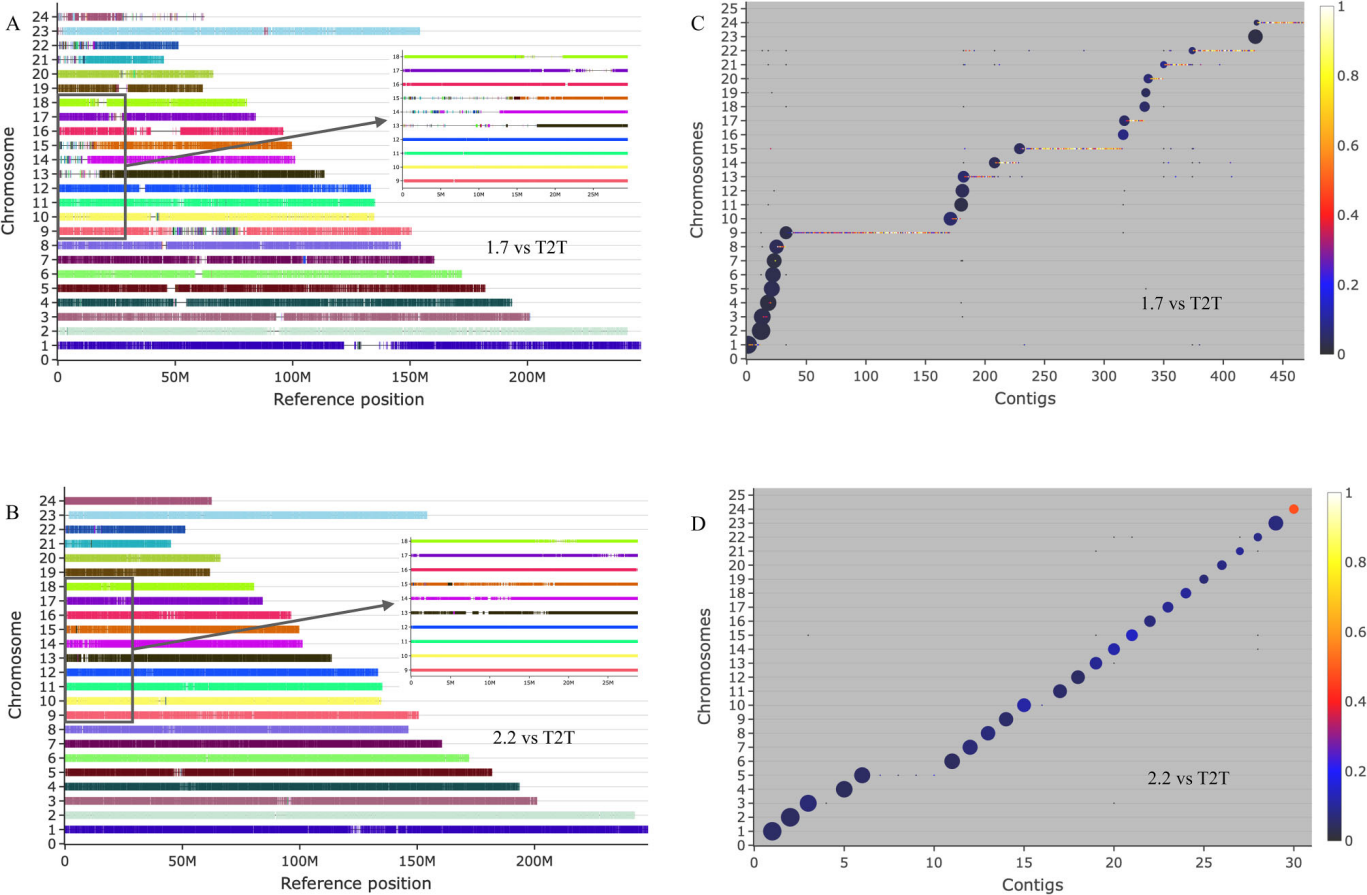
Evaluation of assembly levels while sequencing a given genome. Two versions of a human individual genome, 1.7 (GCA_011064465.1) and 2.2 (GCA_011064465.2), compared to the human T2T assembly (GCF_009914755.1). (**A**, **B**) For each chromosome (*y* axis), alignment blocks are reported as rectangles, coloured according to the contig they belong to, and located at the position they map on the reference chromosome (Mb). Black horizontal lines indicate the chromosome length in the reference assembly. The zoomed areas expand the first ∼30 Mb of chromosomes 9–18. In both plots, *y-*coordinates 23 and 24, respectively, correspond to chromosomes X and Y. (**C, D**) Alignment blocks >10 000 bases are grouped into bubbles located according to the contig (*x* axis) and the chromosome (*y* axis) they belong to. For each bubble, size depends on the total length of the alignment blocks while the colour uses a white-red-blue-black gradient to represent the integrity level, calculated as the fraction of the whole contig length corresponding to the largest block.

The whole genome plots of Fig. 3A and B topographically map similarities and differences onto the various chromosomes. The graph represents chromosomes as an ideogram, where rectangles of each colour indicate blocks from the same contig, located at the position they map on the reference chromosomes. Both assemblies are good quality, as each reference chromosome is mostly covered by one long contig, corresponding to most of its length. However, the representation immediately shows that, in version 1.7, most chromosomes present one or more zones of variously fragmented coverage, enlarged by zooming for chromosomes 13, 14, and 15. Many empty or fragmented regions present in Fig. 3A are filled within one long sequence in version 2.2 (Fig. 3B), which takes advantage of third generation sequencing by PacBio HiFi and Nanopore technologies. The corresponding bubble plots of Fig. 3C and D show how many contigs globally contribute to each chromosome and how fragmented the alignment is. The 1.7 assembly (Fig. 3C) uses >450 contigs to cover the T2T chromosomes: all large contigs show very low continuity, as indicated by the black–blue colour of the bubbles, and over 200 of them are needed only to cover chromosomes 9 and 15. Figure 3D shows that most of these issues are solved in version 2.2, which has a much better correspondence with T2T reference assembly, with only 30 contigs needed to almost completely cover its chromosomes. The large bubbles are mostly located along the main diagonal, with two small interruptions corresponding to chromosomes 3 and 10 and larger one for chromosome 5; a few separated bubbles, typically very small, are also present, which correspond to extra alignment blocks produced when a contig maps on more than one chromosome. Integrity level is also improved, ranging between 0.1 and 0.5, i.e. with larger blocks corresponding to 10%–50% of the contigs mapped on the reference chromosomes (blue–red colours).

Further details about the evaluation of these assemblies are provided in Supplementary Fig. S3, which reports screenshots from the ‘Chromosomes’ tab of ChromoMapperWeb, which gives a chromosome-by-chromosome report in table format and has links to the single-chromosome graphs used to report alignment blocks in a dotplot-like form. In addition, the plots in Supplementary Fig. S4A and B show chromosomes 14 and 15 from version 1.7 aligned with the same chromosomes from version 2.2. The figure highlights how, at the left end of both chromosomes, a number of small contigs, mostly extrachromosomal elements in the first version, have been used to enlarge the chromosome sequence. For chromosome 15, in addition, the plot allows to see that the new version removes a spuriously duplicated sequence, ∼5 Mb long, located around position 30M.

### Rapid analysis of haplotype phased human cell line genomes

**Figure 4.**
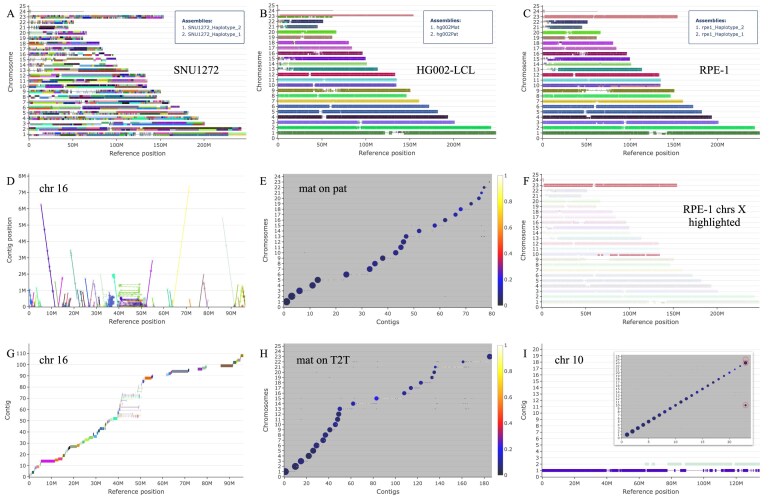
Analysis of human cell line haplotype-phased genomes. (**A**–**C**) Haplotype-phased genomes of three human cell lines, SNU1272 (GCA_040939325.1, GCA_040939355.1), HG002-LCL (GCA_021951015.1, GCA_021950905.1), and RPE-1 (GCA_050656345.1, GCA_050656315.1) mapped onto human T2T genome (GCF_009914755.1). In these as well as the other genome plots in the figure, *y* coordinates 23 and 24, respectively, correspond to chromosomes X and Y. (**D, G**) Mapping of SNU1272 chromosome 16 contigs onto T2T reference chromosome. Bubble plots representing blocks of maternal HG002-LCL haplotype contigs aligned with paternal haplotype chromosomes (**E**) and T2T reference genome (**H**). (**F**) Same plot as in panel (C) with X chromosome sequences highlighted in chromosomes 10 and 23 (chromosome X). (**I**) T2T chromosome 10 aligned with RPE-1 haplotype 1 chromosome 10 sequences (violet) as well as chromosome X (light green). The inset shows blocks, produced by aligning RPE-1 haplotype 1 contigs onto the T2T reference chromosomes, represented as a bubble plot as in panels (E) and (H); red circles highlight three bubbles produced by the alignment of the same RPE-1 contig to chromosomes 10, X, and Y.

ChromoMapperWeb rapidly identifies and highlights the large or even small genomic rearrangements often observed, for example, when assembling cell line genomic sequences. Figure 4A–C shows the genomes of three human cell lines, SNU1272 [36], HG002-LCL [37], and RPE-1 [38], assembled as phased haplotypes and mapped by QUAST alignment onto human T2T genome. For each genome, the two haplotypes are represented as paired tracks for each chromosome.

The plot in Fig. 4A shows the strongly fragmented state of the SNU1272 genome, where each chromosome is covered by many tens of small contigs with several regions left empty (see also Supplementary Fig. S5, where the uncovered regions are plotted in grey for each haplotype). This fragmented pattern may be also due to the haploid mode of assembly, but reflects the highly rearranged nature of the genome, classified as near-triploid but also includes a significant fraction of haploid and diploid chromosomal regions [36]. Chromosome Y (24 on *y* axis) is largely uncovered by both haplotypes, due to the fact that the cells were originally taken from a female subject, as also reported in the source article [36]; the only exception is a small area at left end of chromosome Y, probably corresponding to its pseudo-autosomic region, which is capturing sequences from X chromosome contigs. Plots in Fig. 4D and G highlight the complex alignment pattern produced by sequence rearrangements following a known 10 Mb deletion on chromosome 16, between positions ∼40 and ∼50 Mb [36].

The HG002-LCL lymphoblastoid cell line genome has a standard diploid asset and its alignment (Fig. 4B) shows much better correspondence with the reference assembly. In this case, the genome assembly is much better, with a few very large contigs corresponding to almost full chromosomes, and over 150 much smaller extrachromosomal sequences, which almost fill the remaining regions. The maternal and paternal haplotypes are very similar to each other, as shown in the bubble plot in Fig. 4E where bubbles mostly follow the main diagonal. However, the assembly quality is not the same for all chromosomes, as shown in Fig. 4H, where the maternal haplotype is compared to the human T2T assembly: although all chromosomes mostly correspond to a single contig, chromosomes 1–12 are covered by ∼50 contigs, but over 130 contigs are required to cover the remaining 11 chromosomes.

Figure 4C reports the alignment of the two RPE-1 genome haplotypes to the T2T reference assembly; also in this case the genome has a standard diploid asset. The alignment shows good correspondence with the reference assembly: although, for both haplotypes, each chromosome is covered by one major contig, a major difference is visible in the right half of chromosome 10. Highlighting the upper track contig (Fig. 4F), an unexpected relation becomes clear between chromosomes 10 and X: the right end of T2T chromosome 10 is covered by the same pink contig corresponding to chromosome X. Consistently, Fig. 4I, where coverage of T2T chromosome 10 by the sole haplotype 1 is displayed, shows that at the right end of the reference chromosome there is a combination of blocks from both RPE-1 chromosomes 10 (violet) and X (light green). This ‘anomalous’ relation effectively spots a known feature of the cell line, i.e. the translocation of an extra copy of the long arm of chromosome 10 to the right end of chromosome X [38, 43, 44]. The chromosome translocation is also visible in the bubble plot reported in the inset of the same Fig. 4I: here, all the bubbles are along the main diagonal, the only exception being contig 23 (red circles), which produces two large alignment bubbles, corresponding to chromosomes X and 10, and a much smaller one corresponding to chromosome Y, possibly via the pseudo-autosomic region.

### Genome rearrangements between close species

**Figure 5.**
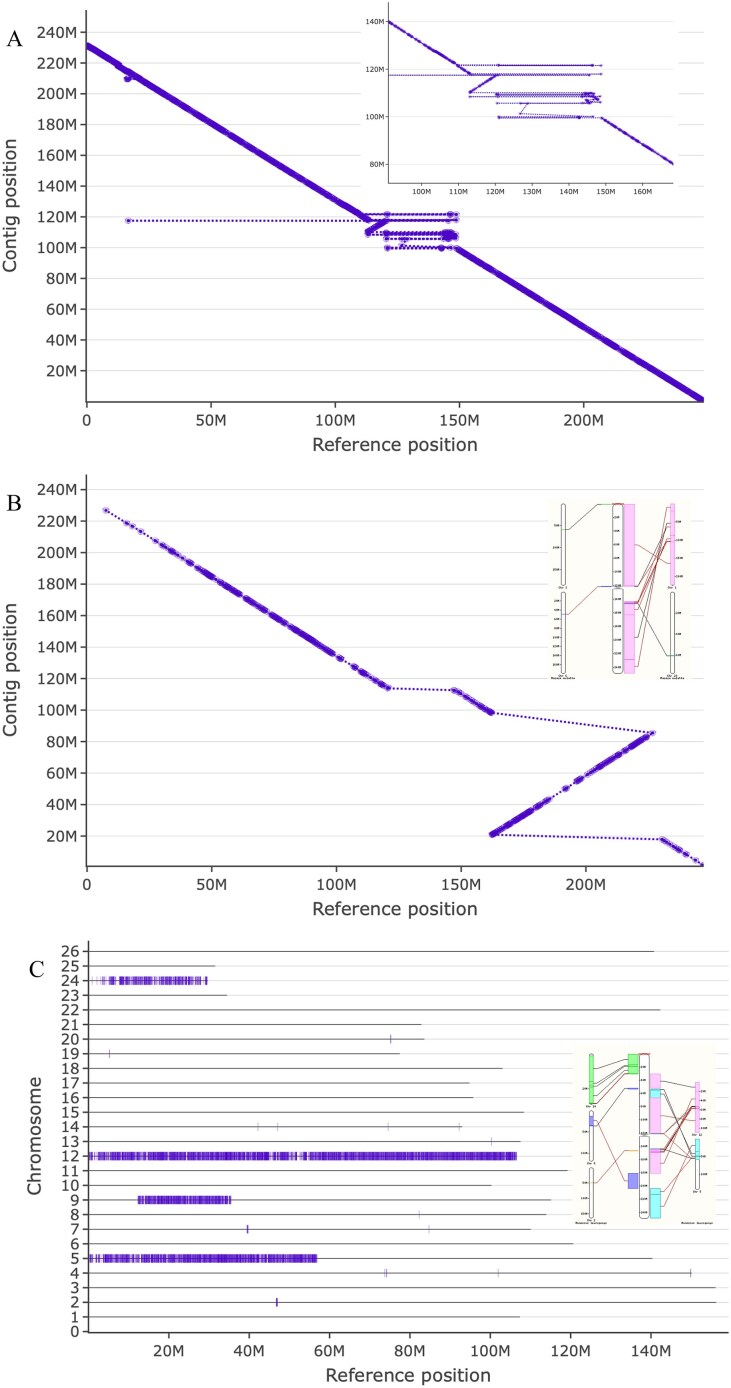
Alignment of genomic sequences from different primates. (**A**–**B**) Dotplot-like representation of chromosome 1 from *P. troglodytes* (GCF_028858775.2) and *M. fascicularis* (GCF_037993035.2) aligned on human T2T genome (GCF_009914755.1). In panel (A), the inset expands the region corresponding to position 110–150 Mb of human chromosome. In panel (B), the inset shows the synteny blocks between human chromosome 1 and macaque genome extracted from Ensembl website. (**C**) Human chromosome 1, from T2T assembly, mapped onto *N. leucogenys* (GCF_006542625.1) assembly, represented within the same genome plot as in Fig. 3. The inset shows synteny blocks reported by Ensembl for the same species.

In addition to intra-species comparisons, ChromoMapperWeb also turns out to be useful when comparing genomes from close species, using the integrated nucmer alignment module. The examples in Fig. 5 show the differences between organization of chromosome 1 in humans and three other primates, *P. troglodytes, M. fascicularis*, and *N. leucogenys*, by using the close species alignment mode (see the ‘Materials and methods’ section). Figure 5A and B shows the alignment of chromosome 1 from *P. troglodytes* (GCF_028858775.2) [41] and *M. fascicularis* (GCF_037993035.2) [42] on T2T human genome: in both cases, the two sequences are mostly collinear with human chromosome 1 sequence, although showing opposite orientation. With *P. troglodytes* chromosome, collinearity spans much of its length, although a relatively long region, corresponding to position 110–150 Mb on human chromosome (i.e. the centromeric region), includes a small inversion and a number of blocks that together do not fully cover the reference sequence (see also zoomed area). A similar gap is also found when aligning *M. fascicularis* sequence, but in this case, the rest of the chromosome is organized in four main alignment regions. The first, ~20 Mb, maps at the right end of human chromosome and, going backwards, is followed by a larger, inverted sequence, corresponding to positions ∼160 to ∼140 Mb on the reference chromosome. The third region includes another small sequence of ∼20 Mb, followed by the previously described gap around the centromere. After that, the alignment restarts and, although less continuous than *P. troglodytes*, it remains collinear with the human sequence (Fig. 5B). The four regions almost exactly correspond to synteny blocks reported by the Ensembl website between human chromosome 1 and macaque genome. Figure 5C reports human chromosome 1 mapped onto *N. leucogenys* assembly (GCF_006542625.1) and shows that in this case, the human sequence is distributed on four different chromosomes: it fully covers chromosomes 12 and 24 and partially chromosomes 5 and 9. Also in this case, the results are consistent with synteny data reported by Ensembl for the same species, displayed in the small inset.

## Discussion

While sequencing a genome, either *de novo* or within a re-sequencing procedure, comparative assembly evaluation is an essential step, either when comparing alternative assembly procedures or when evaluating a genome assembly at different stages or maybe when trying to rapidly highlight gross structural variations, if present. Most alignment analysis tools [10, 13, 45, 46] produce global metrics, which readily distinguish low-quality assemblies from better ones, but issues about how similarity is distributed among chromosomes, where the best assembled regions are located, or which individual contigs and/or alignment blocks are used to build a given chromosome, often are less easy to solve. Alignment analysis tools almost inevitably produce, as a result or sometimes at an intermediate step, long lists of alignment blocks with a lot of detailed information, but to understand where major regions of similarity or main differences are located in the genome, further analysis and/or visualization tools are often required, making the whole process less convenient and sometimes slow.

ChromoMapperWeb was designed to accelerate these tasks by directly reading QUAST output files and folders, as well as other result files in similar format, and quickly identifying and displaying similarities and differences between assemblies within an interactive web environment. By temporarily storing the relevant alignment data on the server, the tool takes a spreadsheet-like approach and visualizes alignment data in tables providing synthetic or detailed reports as well as in dynamically zoomable graphs. With this approach, collinearity between compared sequences, points of inconsistency, discontinuities, repeated regions, and interruption in the assembled sequences are, in most cases, easily spotted, while more detailed analyses are just a few clicks away. Being a web application, ChromoMapperWeb is immediately available on any platform, unlike other tool types that necessarily require installation and configuration on local servers or user machines. The user-friendly graphical interface makes it effective and usable also by researchers with limited computational background. Rapid response, ability to store related data with no previous registration, and quick production of interactive graphics make it also useful as a demonstration tool in academic courses to visualise alignment results.

Within ChromoMapperWeb it is possible to monitor the whole assembly process, all the way from contigs to scaffolds and to chromosomes. At early stages, mapping-assembled contigs onto a reference genome rapidly show how much of it is covered and the degree of integration, either globally or chromosome by chromosome. When scaffolds or full chromosomes become available, they are quickly evaluated by taking advantage of the many table and plot types. The ability to comparatively analyse different alignments on the same reference by using multitrack graphics helps identify common features that remain unchanged between early and late stages of the assembly process. The multitrack feature is also used to analyse paired assemblies corresponding to haplotypes produced by phased assembly or dedicated haplotagging tools, a datatype more and more common when sequencing individual genomes or genomic DNA extracted from cultured cells.

ChromoMapperWeb includes data-analysis code from its companion command line tool, ChromoMapper [23], and makes it available through an easy-to-use graphical interface in an environment where most re-calculations are ready in seconds and all further re-sizing is carried out locally within the browser. ChromoMapperWeb can store many different experiments and make them available within just a few clicks; it also adds the option to upload assembled genome sequences and align them by using nucmer, a handy feature, especially for small datasets that do not require long running times. The web application quickly produces tables containing chromosome-by-chromosome summaries of genome coverage, as well as detailed information on single chromosomes or contigs; graphical alignment representations are used to visualize alignment blocks in detail. Within ChromoMapperWeb, most output tables and plots are rapidly re-calculated and re-displayed, after changing parameters through the always-available forms. While viewing any table, further custom-built graphs may be produced on the spot, by plotting any data column against any other. Supplementary Table S1 compares ChromoMapperWeb and its command line counterpart, ChromoMapper, with other tools that, over time, similarly tried to tackle the many issues deriving from genome size, especially in mammals, sequence variability, and the need to adapt to the ever-growing number of sequence-dataset types produced by different experimental procedures and instrumentations. The tools in the last three columns (IGV, JBrowse2, and CGV) have been kept separate as, being full featured genome browsers, they are typically focused on genomic data display, a task where they excel, rather than on performing alignment analysis [21, 22, 47]. This group includes CGV [22], effectively used by the NCBI site to produce beautifully interactive resizable genome views, but primarily directed to display a selection of pre-calculated genome alignments. The other assembly analysis tools, in contrast, mostly support user-provided assemblies and generate reports focused on metrics describing an assembly or alignment and produce graphic representations such as dot-plots, usually via static plots. A notable exception is QUAST, which uses the linked Icarus genome viewer to dynamically display alignment blocks. Unlike most tools, ChromoMapperWeb supports the analysis of pre-calculated alignments produced by other tools, including QUAST itself, and also user-provided assemblies, through an embedded alignment tool (nucmer); post-processing of alignment data includes evaluating them at chromosome/scaffold level as well as contig by contig and even at the level of each individual block (Supplementary Table S1). Results are rapidly made available through detailed tables organized by chromosome, contig, extra-chromosomal sequences, and alignment blocks.

In ChromoMapperWeb, execution times are indeed short, as most graphs and tables are produced within one or a few seconds, even for genomes as large as mammalians, except for the relatively rare cases when an alignment is fragmented in a very large number of contigs and/or blocks (Supplementary Table S2). But the idea behind ‘rapid’ analysis also includes the advantages of accessing pre-loaded data through a web interface, allowing immediate access to plots and tables, without wasting time with possibly complex command lines and long files paths. Quick modification of plot and table parameters in dialog forms, followed by fast re-calculation, also helps towards ‘rapid’ analysis of the data; even importing uploaded data is typically done in a few seconds. Finally, the possibility to keep many alignment results readily available online and organized in parallel projects certainly helps in making the tool easy and convenient to use and eventually rapid.

Performance, of course, also depends on the used hardware. In ChromoMapperWeb, a relevant feature is that most graphic calculation is offloaded to the client and therefore performance also depends on the quality of the used hardware. This feature may sometimes become a limitation with less powerful client machines, especially in graph production; in this case the analysis should be restricted to simpler assemblies, in order to maintain a reasonable interactivity and to allow the production of more complex graphics. However, as the application works on alignment blocks, most current machines can easily display full genomes without problems, even when the hardware is not optimal. On the other hand, when using good performance workstations, the application can strongly benefit from the availability of graphic resources and fast network connections, which, allowing rapid data transfer and display of complex graphics, make it very easy to work with large number of blocks or to perform comparative analyses, involving many different assemblies on the same reference.

## Supplementary Material

gkag506_Supplemental_File

## Data Availability

ChromoMapperWeb is freely available at https://chromomapperweb.ceinge.unina.it/. No registration is required. All the sequences used within the article are freely available within public repositories as described under Material and Methods.
