# Supplementary material for: ChromoMapperWeb: evaluate genome alignments and track assembly steps within an interactive graphic environment
Source: Nucleic Acids Res. 2026 May 21;54(W1):W85–95. doi: 10.1093/nar/gkag506 (PMC13355067; doi:10.1093/nar/gkag506)
Supplement: gkag506_Supplemental_File [file gkag506_supplemental_file.pdf]

SUPPLEMENTARY MATERIAL

ChromoMapperWeb: evaluate genome alignments and track assembly steps within an interactive graphic environment

Elvira Toscano[1,2], Elena Cimmino[1,2], Angelo Boccia[1], Leandra Sepe[2] and Giovanni Paolella[2,1,*]
[1] CEINGE - Biotecnologie Avanzate "Franco Salvatore", Via Gaetano Salvatore, 486, 80145, Napoli, Italy
[2] Dipartimento di Medicina Molecolare e Biotecnologie Mediche, Università degli Studi di Napoli Federico II, Via Sergio Pansini, 5, 80131, Napoli, Italy
[*] To whom correspondence should be addressed. Email: giovanni.paolella@unina.it

Supplementary Text 1. Production of alignment data, processing and result generation

Management of nucmer alignment tasks

The execution of nucmer-based alignment tasks within the *ChromoMapperWeb* is managed through an asynchronous queue system. The management process periodically runs while the application is in use and monitors active or pending executions. Each alignment task is represented by a seqAligner object, initially placed in a waiting state. The transition to the running state occurs automatically when sufficient computational resources are available. Resource availability is determined through a "points" system that limits the number of parallel executions based on their computational complexity. Each alignment class is associated with a cost in points, proportional to the expected computational load; the system imposes a maximum limit on the number of points that can be used simultaneously. New alignments are started only if their point cost is compatible with the remaining resources. Queued requests associated with active users are served with priority over the others. Completed runs are moved to the to-be-imported state and later imported into the application storage system. In case of errors or incomplete results, the run is moved to a retry queue, to allow further analysis or reruns.

Alignment block pre-processing and analysis

A chromoExp object organizes the alignment results in different tables by using objects of type sheet. A blocksSheet object is used to produce a large table containing the imported blocks; it can be displayed or also used as a base to produce other sheets. Within the object, the stored alignment blocks are organized according to the chromosome on which they map and for each of them, a set

of extra parameters are calculated, describing block features, block end annotations and number of related blocks.

Block statistics are computed within the BlockstatsSheet, which organises blocks according to their length in different categories (above or below a length threshold or between two lengths) and, for each group of blocks, calculates the various statistical parameters. The same block data are also used for building a bubble plot, used to show how blocks contribute to relate each contig with the chromosome(s) on which it maps.

Building reference chromosomes by organising mapped blocks

The information about reference genome coverage is calculated in chromomapSheet object: blocks larger than a user-defined length and mapping on the reference sequences classified as chromosomes are re-ordered according to chromosome and start position they map on, after adding further parameters. The same ordered blocks are also used by chromotableSheet to provide a rapid chromosome-by-chromosome view of the major features of the reference genome coverage and by graphic functions to build a number of graphical representations of the alignment blocks at single chromosome or whole genome level. Finally, chromostatsSheet provides synthetic statistical evaluation of the reference chromosome coverage. An extrachromoSheet object is used to operate on extrachromosomal sequences, i.e. reference sequences not corresponding to known chromosomes.

Assembled contig evaluation

Much information about assembly contigs is calculated, as described for chromosomes, by using sheet objects. Blocks, ordered according to the contig and the position they map on, are listed within contigmapSheet and analysed within contigsSheet, to rapidly provide a view of contig coverage. Statistical evaluation of contig features is calculated by the contigstatsSheet object.

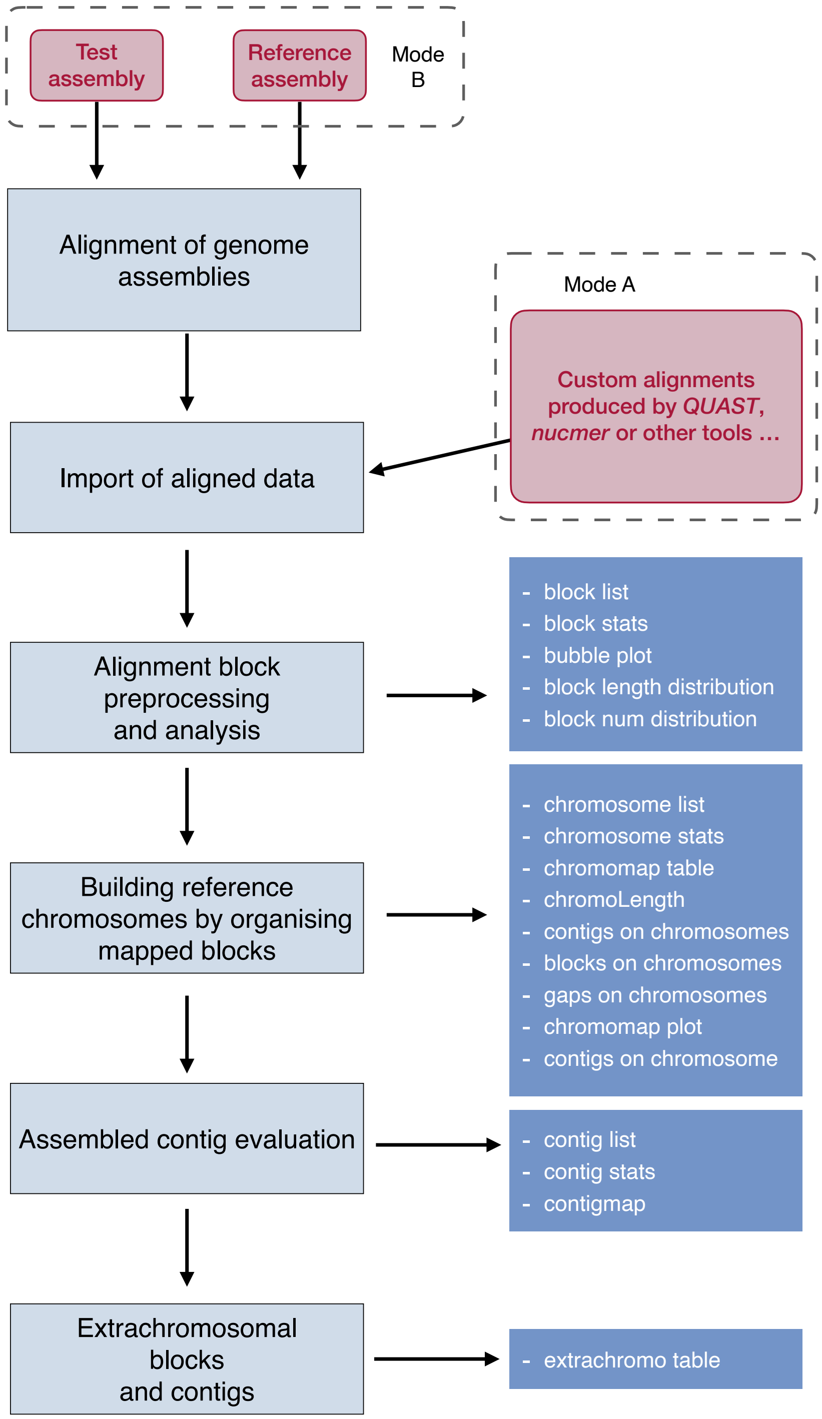

Supplementary Figure 1. Workflow schema. Overview of the analysis steps (light blue boxes) performed within *ChromoMapperWeb*, from input data (red boxes) to results displayed within web pages (blue boxes). Mode A and B refer to the two different usage modes, starting from pre-calculated alignments or aligning assemblies within the tool (see Materials and Methods).

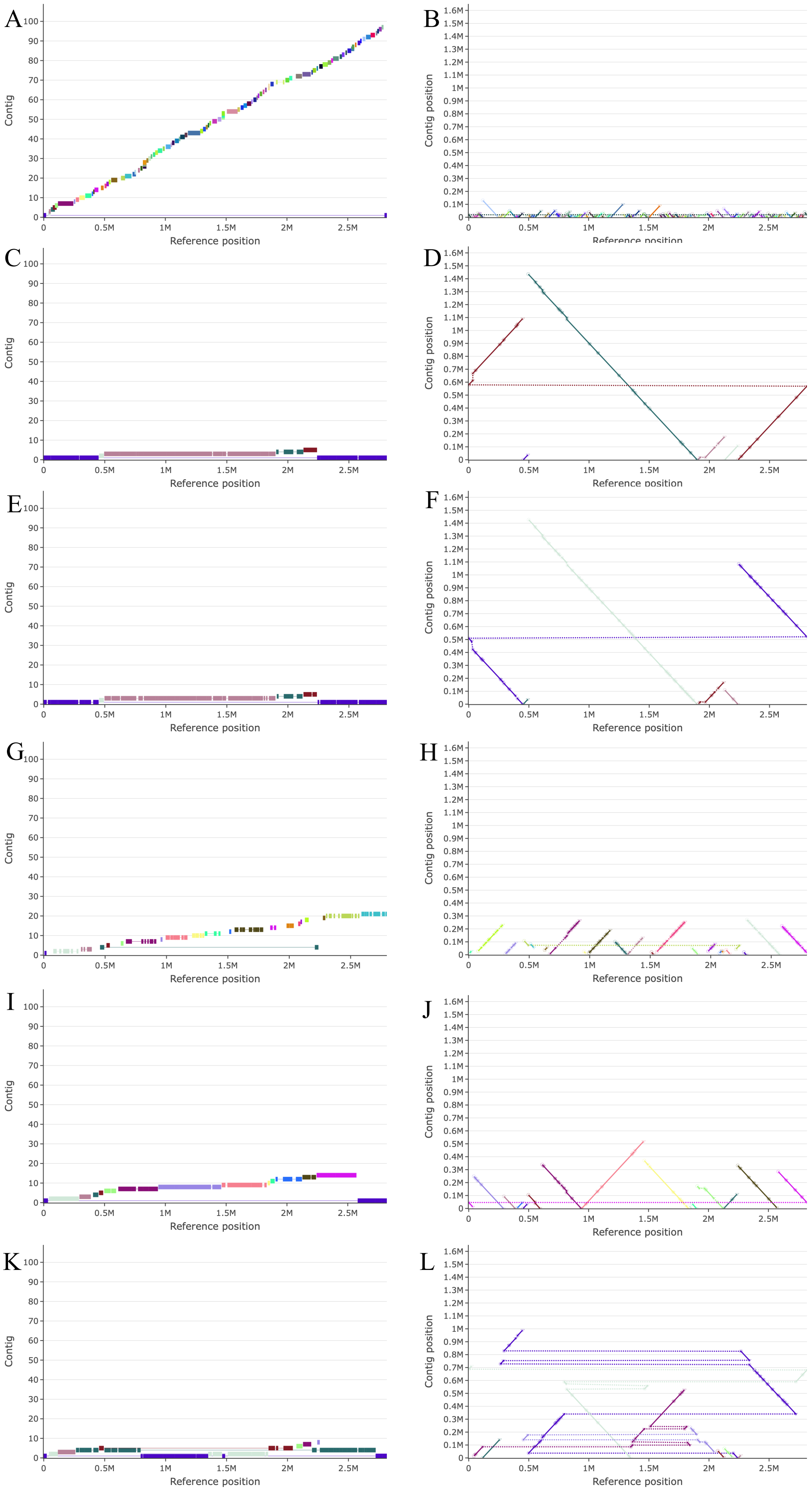

A
B
C
D
E
F
G
H
I
J
K
L
Contig
Contig position
Reference position
0
10
20
30
40
50
60
70
80
90
100
0.1M
0.2M
0.3M
0.4M
0.5M
0.6M
0.7M
0.8M
0.9M
1M
1.1M
1.2M
1.3M
1.4M
1.5M
1.6M
0.5M
1M
1.5M
2M
2.5M

Supplementary Figure 2. Alignment of assemblies produced by different tools from a set of *S. aureus* reads. Assemblies produced by *ABySS* (**A-B**), *ALLPATHS-LG* (**C-D**), *Bambus2* (**E-F**), *SGA* (**G-H**), *SOAPdenovo* (**I-J**) and *Velvet* (**K-L**) aligned with *S. aureus* genome sequence (GCF_000013425.1) used as reference. In plots (**A**,**C**,**E**,**G**,**I**,**K**), the alignment blocks are displayed as coloured rectangles, located at the position on which they map on the reference chromosome (Mbases). Blocks from the same contig are reported at the same level on the y axis; thinner lines are used to connect the contiguous blocks of the same contig which map on different positions on the chromosome. In plots (**B**,**D**,**F**,**H**,**J**,**L**), graphs are dotplot-like representations, where blocks are reported as segments placed at their reference/contig position (Mbases) and tagged with start (circles) and stop (triangles) symbols. Dotted lines are used to connect not contiguous blocks on the reference chromosome. The *ABySS* tool produces the least integrated assembly, with almost 100 small contigs needed to cover the whole genome. *ALLPATHS-LG* and *Bambus2*, instead, produce the most continuous assemblies, both composed of only five contigs, two of them (1.1 and 1.4 Mbases) long enough to cover over 70% of the reference genome. In both cases, two halves of the same contig map at the beginning and end of the reference genome; due to the circular nature of the S. aureus genome, they only apparently represent as separate blocks, as they are connected through the ends. Contig alignment includes a different number of blocks, 67 for *Bambus2* and 37 for *ALLPATHS-LG*, but their organization is very similar, suggesting that the small differences between the two assemblies and the reference sequence are likely due to real differences among the compared genomes rather than errors in the assembly procedure. The assemblies by SGA and *SOAPdenovo* are intermediate assemblies, requiring 21 and 14 contigs to cover the reference genome. Finally, the assembly by *Velvet* consists of 8 longer contigs, but presents many sequence relocations, possibly pointing to a higher error level during the assembly steps.

Block stats | Blocks | Contig stats | Contigs | Contigmap | Chromo stats | Chromosomes | Chromomap | Extrachromo

A

refID	S1	E1	Coverage	nContigs	L98	L95	L90	L50	IDY	nBlocks	Length	RefLen	DiffLen	Map	Contigs
Chr1	95,994	248,382,082	91.53	15	15	15	1	1	99.46	571	227,338,588	248,387,328	21,048,740		
Chr2	4,505	242,651,388	98.06	2	1	1	1	1	99.52	654	237,981,209	242,696,752	4,715,543		
Chr3	2,646	201,101,183	97.36	6	6	1	1	1	99.49	749	195,792,198	201,105,948	5,313,750		
Chr4	3,238	193,435,733	96.83	4	4	1	1	1	99.49	651	187,430,482	193,574,945	6,144,463		
Chr5	2,297	182,043,893	97.29	3	3	1	1	1	99.48	556	177,118,948	182,045,439	4,926,491		
Chr6	2,876	172,082,869	97.77	3	3	1	1	1	99.60	581	168,283,818	172,126,628	3,842,810		
Chr7	25,247	160,563,866	97.86	5	5	1	1	1	99.44	708	157,132,612	160,567,428	3,434,816		
Chr8	2,020	146,254,006	97.39	9	9	1	1	1	99.31	338	142,434,938	146,259,331	3,824,393		
Chr9	160,926	150,561,315	82.64	140	140	140	140	1	99.01	841	124,462,646	150,617,247	26,154,601		
Chr10	3,513	134,755,095	97.69	9	9	1	1	1	99.44	502	131,646,780	134,758,134	3,111,354		
Chr11	1,578	135,125,192	97.00	4	4	1	1	1	99.38	472	131,073,544	135,127,769	4,054,225		
Chr12	20,003	133,324,512	97.60	3	3	1	1	1	99.49	391	130,130,087	133,324,548	3,194,461		
Chr13	41,558	113,563,140	85.68	34	34	34	34	1	99.06	233	97,308,798	113,566,686	16,257,888		
Chr14	516,579	101,159,990	90.42	27	27	27	10	1	99.34	472	91,473,801	101,161,492	9,687,691		
Chr15	133,429	99,654,864	88.62	93	93	93	93	1	99.20	521	88,400,564	99,753,195	11,352,631		
Chr16	3,510	96,254,492	82.51	1	1	1	1	1	99.26	261	79,484,410	96,330,374	16,845,964		
Chr17	29,559	84,266,474	93.45	17	17	17	1	1	99.13	221	78,756,469	84,276,897	5,520,428		
Chr18	214,277	80,539,499	93.52	5	5	5	1	1	99.41	186	75,326,539	80,542,538	5,215,999		
Chr19	10,985	61,703,983	94.05	4	4	4	1	1	99.12	207	58,036,441	61,707,364	3,670,923		
Chr20	10,449	66,207,088	94.18	14	14	14	1	1	99.26	278	62,356,091	66,210,255	3,854,164		
Chr21	3,009	45,086,177	83.84	33	33	33	33	1	98.52	183	37,804,576	45,090,682	7,286,106		
Chr22	751,740	51,322,071	82.49	66	66	66	66	1	98.48	360	42,337,163	51,324,926	8,987,763		
ChrX	5,675	154,234,957	96.54	2	2	1	1	1	99.40	409	148,915,831	154,259,566	5,343,735		
ChrY	5,691	62,170,497	43.28	42	42	42	42	42	99.13	144	27,035,067	62,460,029	35,424,962		

Block stats | Blocks | Contig stats | Contigs | Contigmap | Chromo stats | Chromosomes | Chromomap | Extrachromo

B

refID	S1	E1	Coverage	nContigs	L98	L95	L90	L50	IDY	nBlocks	Length	RefLen	DiffLen	Map	Contigs
Chr1	116,315	248,383,452	95.14	1	1	1	1	1	99.27	496	236,305,244	248,387,328	12,082,084		
Chr2	1	242,696,752	99.38	1	1	1	1	1	99.32	196	241,197,255	242,696,752	1,499,497		
Chr3	2,297	201,102,610	99.32	3	1	1	1	1	99.43	156	199,729,815	201,105,948	1,376,133		
Chr4	3,238	193,573,144	98.88	1	1	1	1	1	99.51	221	191,397,955	193,574,945	2,176,990		
Chr5	2,294	182,044,072	98.41	5	1	1	1	1	99.27	135	179,157,841	182,045,439	2,887,598		
Chr6	2,876	172,123,911	98.81	1	1	1	1	1	99.49	173	170,073,418	172,126,628	2,053,210		
Chr7	3,201	160,565,235	98.75	1	1	1	1	1	99.47	254	158,554,712	160,567,428	2,012,716		
Chr8	1	146,259,331	99.05	1	1	1	1	1	99.45	130	144,875,973	146,259,331	1,383,358		
Chr9	3,624	150,614,887	94.48	1	1	1	1	1	98.88	736	142,308,363	150,617,247	8,308,884		
Chr10	1,710	134,755,089	98.73	2	1	1	1	1	99.21	146	133,040,664	134,758,134	1,717,470		
Chr11	110	135,125,192	99.26	1	1	1	1	1	99.29	107	134,124,604	135,127,769	1,003,165		
Chr12	1	133,322,343	98.84	1	1	1	1	1	99.42	136	131,782,067	133,324,548	1,542,481		
Chr13	1	113,563,599	96.46	2	2	1	1	1	99.18	272	109,549,478	113,566,686	4,017,208		
Chr14	1	101,160,184	96.87	2	2	1	1	1	99.24	154	97,999,599	101,161,492	3,161,893		
Chr15	1	99,750,303	97.51	4	4	1	1	1	99.42	328	97,270,942	99,753,195	2,482,253		
Chr16	2,250	96,328,075	95.86	1	1	1	1	1	99.51	394	92,341,972	96,330,374	3,988,402		
Chr17	2,091	84,276,475	97.60	1	1	1	1	1	99.16	152	82,256,674	84,276,897	2,020,223		
Chr18	1	80,539,927	97.31	1	1	1	1	1	99.35	159	78,373,490	80,542,538	2,169,048		
Chr19	1	61,707,364	94.90	1	1	1	1	1	98.76	166	58,558,992	61,707,364	3,148,372		
Chr20	277	66,208,069	97.43	1	1	1	1	1	99.29	140	64,509,860	66,210,255	1,700,395		
Chr21	3,010	45,086,177	97.02	3	3	1	1	1	98.64	128	43,745,492	45,090,682	1,345,190		
Chr22	4,348	51,322,262	96.64	4	4	1	1	1	99.09	194	49,602,442	51,324,926	1,722,484		
ChrX	108,535	154,257,238	98.72	1	1	1	1	1	99.45	174	152,287,906	154,259,566	1,971,660		
ChrY	1	62,458,777	99.96	1	1	1	1	1	100.00	20	62,435,133	62,460,029	24,896		

Supplementary Figure 3. Chromosome coverage evaluation while sequencing a given genome. Two versions of a human individual genome, (**A**) 1.7 (GCA_011064465.1) and (**B**) 2.2 (GCA_011064465.2), are compared to the human telomere-to-telomere (T2T) assembly (GCF_009914755.1). For each alignment, the *Chromosomes* table calculated within *ChromoMapperWeb* is reported.

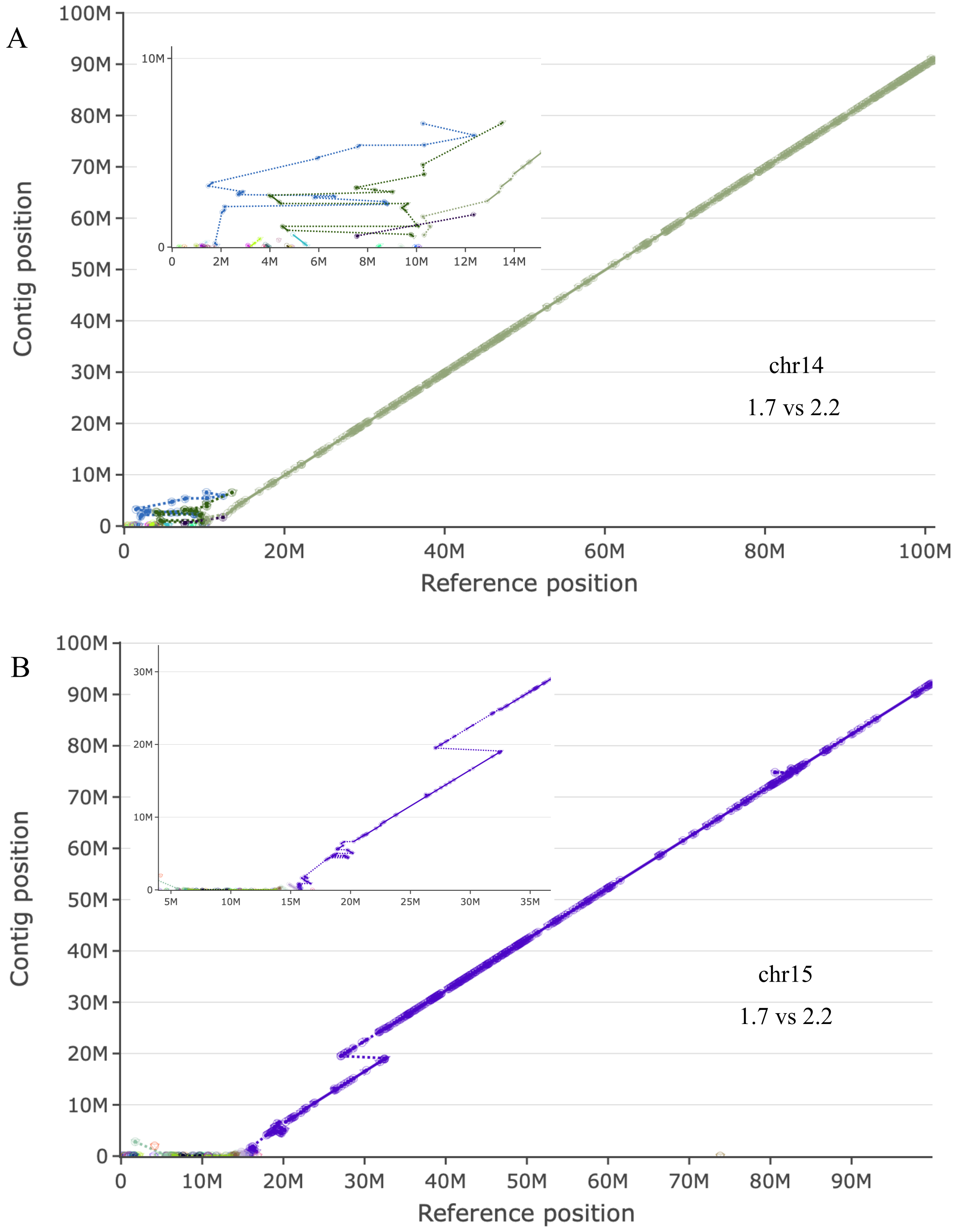

Supplementary Figure 4. Evaluation of assembly levels while sequencing a given genome. Dotplot-like representation of chromosomes 14 and 15 from the alignment of human individual genome, version 1.7 (GCA_011064465.1) onto 2.2 one (GCA_011064465.2). The enlarged panels expand the regions between 0-15 and 4-35 Mbases on chromosomes 14 and 15 of the reference genome.

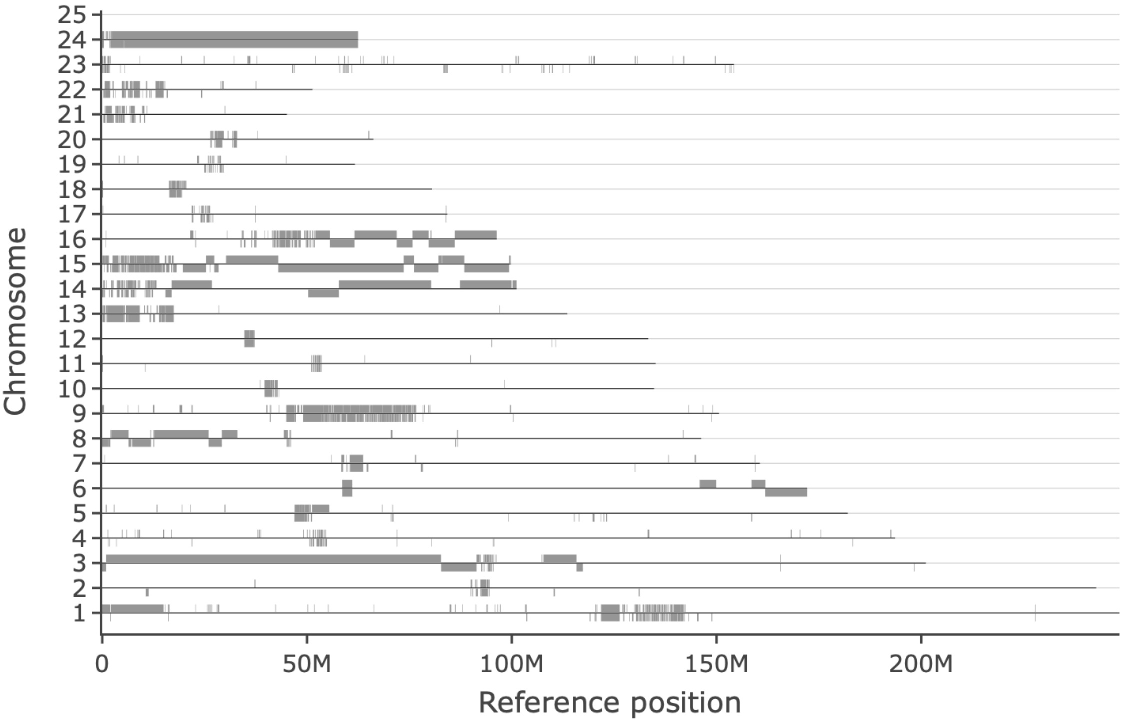

Supplementary Figure 5. Gap plot for SNU1272 haploid genome. Haploid genomes of the human cell line SNU1272 (GCA_040939325.1, GCA_040939355.1), mapped onto human T2T genome (GCF_009914755.1). Uncovered regions are plotted in grey for each haplotype. Haploid regions in chromosomes 3, 6, 8, 14, 15 and 16 are readily guessed from the alternated distribution of the gaps on the two haplotypes.

		Mummer package	Simple-Synteny	Synteny Portal	QUAST/ Icarus	DiGAlign	Chromo Mapper	Chromo Mapper Web	IGV	JBrowse 2	CGV (NCBI)
**year**		2004-18	2016	2016	2013-23	2024	2026	2026	2011	2023	2024
**locally installed tool**		√			√		√		√	√	
**public server**			√	√	√	√		√	√		√
**Genome size:**	**small (bacteria)**		small		small	small	small	small			
	**large (mammals)**	large		large	large		large	large	large	large	large
**Assembly data**	**pre-installed on server**			√				samples			√
	**custom (user-provided)**	√	√		√	√	√	√	√	√	
**Assembly mapping**	**independently pre-calculated**						Quast, nucmer	Quast, nucmer			
	**executed within tool**	nucmer (native)	Blast		Minimap 2	Blast		nucmer			
**Mapping level**	**gene**		√			√					
	**block**	√			√		√	√			
	**contig**	√			√	√	√	√			
	**scaffold/ chromosome**	√		√	√	√	√	√	√	√	√
**Graphical visualisation and analysis**	**single-chromosome dot-plots**	√				√	√	√		√	√
	**alignment-block organization on chromosomes**						√	√			
	**ideograms for whole genome coverage**						√	√			
	**zooming via dynamically modified plots**						+++	+++			
	**comparative genome viewer**		+	+	++		++	++	+++	+++	++++
**Assembly and alignment evaluation**	**assembly statistics**				√			√			
	**global alignment statistics**				√		√	√			
	**analysis of alignment blocks**				√		√	√			
**Parameters and numerical analyses**	**genome coverage per-chromosome**						√	√			
	**block by block chromosome coverage**						√	√			
	**block by block contig mapping**						√	√			
	**extra-chromosomal sequence coverage**						√	√			
	**multiple alignment analysis**			√	√	√		√		√	
**ref**		(1,2)	(3)	(4)	(5,6)	(7)	(8)	-	(9)	(10)	(11)

Supplementary Table 1. Comparison of *ChromoMapperWeb* with other tools. The table compares *ChromoMapperWeb* and its command line counterpart, *ChromoMapper*, with other tools, which, over time, tried to tackle the many issues deriving from genome size, especially with large mammalian genomes, sequence variability and the need to adapt to ever-growing type of sequence datasets produced by different experimental procedures and instrumentations. The tools in the last three columns (IGV, JBrowse2 and CGV) have been kept separate as, being full featured genome browsers, they excel in terms of genomic data display and are typically focused on viewing results rather than on performing analysis of alignments.

Feature	Reference	GCA_043295415.1	GCF_009914755.1 (T2T)	GCF_009914755.1 (T2T)
	Assembly	*B. impatiens (SOAPdenovo)*	*H. sapiens -* GRCh38.p14	*H. sapiens -* SNU1272Hap1
Genome size		266,572,400	3,117,275,501	2,911,075,934
Number of ref chromosomes		18	24	24
Number of contigs		25,528	705	2,678
Number of blocks		8,793	7,920	29,999
Producing Contigs table		1.088	1.130	6.255
Producing Chromosomes table		0.400	0.670	1.786
Producing Block stats table		1.047	1.262	5.805
Producing Contig stats table		1.021	0.995	5.681
Producing Chromo stats table		0.311	0.363	1.248
Producing Chromomap table for chr 1		0.382	0.836	1.614
Producing Chromomap table for chr 18		0.390	0.545	1.319
Producing Contigmap table for chr 1		0.460	0.466	1.599
Producing Contigmap table for chr 18		0.510	0.395	1.307
Producing Bubble plot (>10000)		1.390	3.096	10.596
Producing ChromoLenght plot		0.426	0.338	1.236
Producing Contigs on chromosomes plot		1.993	1.375	2.737
Producing Blocks on chromosomes plot		2.163	1.098	4.634
Producing Gaps on chromosomes plot		0.626	0.603	1.709
Producing Contigs on chromosome plot for chr 1		0.608	1.267	1.542
Producing Contigs on chromosome plot for chr 18		0.528	0.431	1.422
Producing Chromomap plot for chr 1		0.816	0.557	1.578
Producing Chromomap plot for chr 18		0.479	0.374	1.812

Supplementary Table 2. Execution times for different assembly analyses. The table reports the analysis of a *B. impatiens* assembly (12) produced by using *SOAPdenovo* and mapped on the reference genome, GCA_043295415.1, the human genome GHCR38.p14, cell line SNU1272 genome assembly (13); the last two are mapped on the human T2T genome assembly (GCF_009914755.1). Execution times are in seconds and were obtained using as client an Intel MacBook Pro.

References

1. Kurtz,S., Phillippy,A., Delcher,A.L., Smoot,M., Shumway,M., Antonescu,C. and Salzberg,S.L. (2004) Versatile and open software for comparing large genomes. Genome Biology, 5, R12.
https://doi.org/10.1186/gb-2004-5-2-r12

2. Marçais,G., Delcher,A.L., Phillippy,A.M., Coston,R., Salzberg,S.L. and Zimin,A. (2018) MUMmer4: A fast and versatile genome alignment system. PLoS Comput Biol, 14, e1005944.
https://doi.org/10.1371/journal.pcbi.1005944
http://www.ncbi.nlm.nih.gov/pmc/articles/PMC5802927

3. Veltri,D., Wight,M.M. and Crouch,J.A. (2016) SimpleSynteny: a web-based tool for visualization of microsynteny across multiple species. Nucleic Acids Res, 44, W41–W45.
https://doi.org/10.1093/nar/gkw330
http://www.ncbi.nlm.nih.gov/pmc/articles/PMC4987899

4. Lee,J., Hong,W.-Y., Cho,M., Sim,M., Lee,D., Ko,Y. and Kim,J. (2016) Synteny Portal: a web-based application portal for synteny block analysis. Nucleic Acids Res, 44, W35-40.
https://doi.org/10.1093/nar/gkw310
http://www.ncbi.nlm.nih.gov/pmc/articles/PMC4987893

5. Gurevich,A., Saveliev,V., Vyahhi,N. and Tesler,G. (2013) QUAST: quality assessment tool for genome assemblies. Bioinformatics, 29, 1072–1075.
https://doi.org/10.1093/bioinformatics/btt086
http://www.ncbi.nlm.nih.gov/pmc/articles/PMC3624806

6. Mikheenko,A., Saveliev,V., Hirsch,P. and Gurevich,A. (2023) WebQUAST: online evaluation of genome assemblies. Nucleic Acids Res, 51, W601–W606.
https://doi.org/10.1093/nar/gkad406
http://www.ncbi.nlm.nih.gov/pmc/articles/PMC10320133

7. Nishimura,Y., Yamada,K., Okazaki,Y. and Ogata,H. (2024) DiGAlign: Versatile and Interactive Visualization of Sequence Alignment for Comparative Genomics. Microbes Environ, 39, ME23061.
https://doi.org/10.1264/jsme2.ME23061
http://www.ncbi.nlm.nih.gov/pmc/articles/PMC10982109

8. Toscano,E., Cimmino,E., Boccia,A., Sepe,L. and Paolella,G. (2026) ChromoMapper: a new tool to quickly compare large genome assemblies. Bioinformatics Advances, 10.1093/bioadv/vbag005.
https://doi.org/10.1093/bioadv/vbag005

9. Thorvaldsdóttir,H., Robinson,J.T. and Mesirov,J.P. (2013) Integrative Genomics Viewer (IGV): high-performance genomics data visualization and exploration. Brief Bioinform, 14, 178–192.
https://doi.org/10.1093/bib/bbs017
http://www.ncbi.nlm.nih.gov/pmc/articles/PMC3603213

10. Diesh,C., Stevens,G.J., Xie,P., De Jesus Martinez,T., Hershberg,E.A., Leung,A., Guo,E., Dider,S., Zhang,J., Bridge,C., et al. (2023) JBrowse 2: a modular genome browser with views of synteny and structural variation. Genome Biol, 24, 74.
https://doi.org/10.1186/s13059-023-02914-z
http://www.ncbi.nlm.nih.gov/pmc/articles/PMC10108523

11. Rangwala,S.H., Rudnev,D.V., Ananiev,V.V., Oh,D.-H., Asztalos,A., Benica,B., Borodin,E.A., Bouk,N., Evgeniev,V.I., Kodali,V.K., et al. (2024) The NCBI Comparative Genome Viewer (CGV) is an interactive visualization tool for the analysis of whole-genome eukaryotic alignments. PLoS Biol, 22, e3002405.
https://doi.org/10.1371/journal.pbio.3002405
http://www.ncbi.nlm.nih.gov/pmc/articles/PMC11101090

12. Salzberg,S.L., Phillippy,A.M., Zimin,A., Puiu,D., Magoc,T., Koren,S., Treangen,T.J., Schatz,M.C., Delcher,A.L., Roberts,M., et al. (2012) GAGE: A critical evaluation of genome assemblies and assembly algorithms. Genome Res, 22, 557–567.
https://doi.org/10.1101/gr.131383.111
http://www.ncbi.nlm.nih.gov/pmc/articles/PMC3290791

13. Han,H., Lee,H.H., Kim,M.G., Shin,Y.S., Chung,J.S. and Kim,J. (2025) Genome assembly resources of genitourinary cancers for chromosomal aberration at the single nucleotide level. Sci Data, 12, 550.
https://doi.org/10.1038/s41597-025-04801-7
